# Supplementary material for: A cholesterol-responsive hepatic tRNA-derived small RNA regulates cholesterol homeostasis and atherosclerosis development
Source: Nat Commun. 2025 Dec 15;16:11043. doi: 10.1038/s41467-025-67387-z (PMC12706008; doi:10.1038/s41467-025-67387-z)
Supplement: Supplementary file 1 — Supplementary Information [file 41467_2025_67387_MOESM1_ESM.pdf]

Supplementary Information for

A cholesterol-responsive hepatic tRNA-derived small RNA regulates cholesterol homeostasis and atherosclerosis development

Xiuchun Li,[1] Rebecca Hernandez,[1] Xudong Zhang,[2,3] Sijie Tang,[1] Xiaohong Yuan,[4] Jing Wu,[4] Kathy Pham,[1] Hukam C. Rawal,[5] Erica C. Heinrich,[1] Shenglong Zhang,[4,6,7] Qi Chen,[2,3] Tong Zhou,[5] and Changcheng Zhou[1,*]

[1] Division of Biomedical Sciences, School of Medicine, University of California, Riverside, CA, USA
[2] Molecular Medicine Program, Department of Human Genetics, University of Utah School of Medicine, Salt Lake City, UT, USA
[3]Division of Urology, Department of Surgery University of Utah School of Medicine, Salt Lake City, UT, USA
[4]Department of Biological and Chemical Sciences, New York Institute of Technology, New York, NY, USA
[5]Department of Physiology and Cell Biology, University of Nevada, Reno School of Medicine, Reno, NV, USA
[6]Department of Chemistry, University at Albany, State University of New York, Albany, NY, USA
[7]The RNA Institute, University at Albany, State University of New York, Albany, NY, USA

This PDF file includes:

Supplementary Figure 1-8
Supplementary Table 1-3

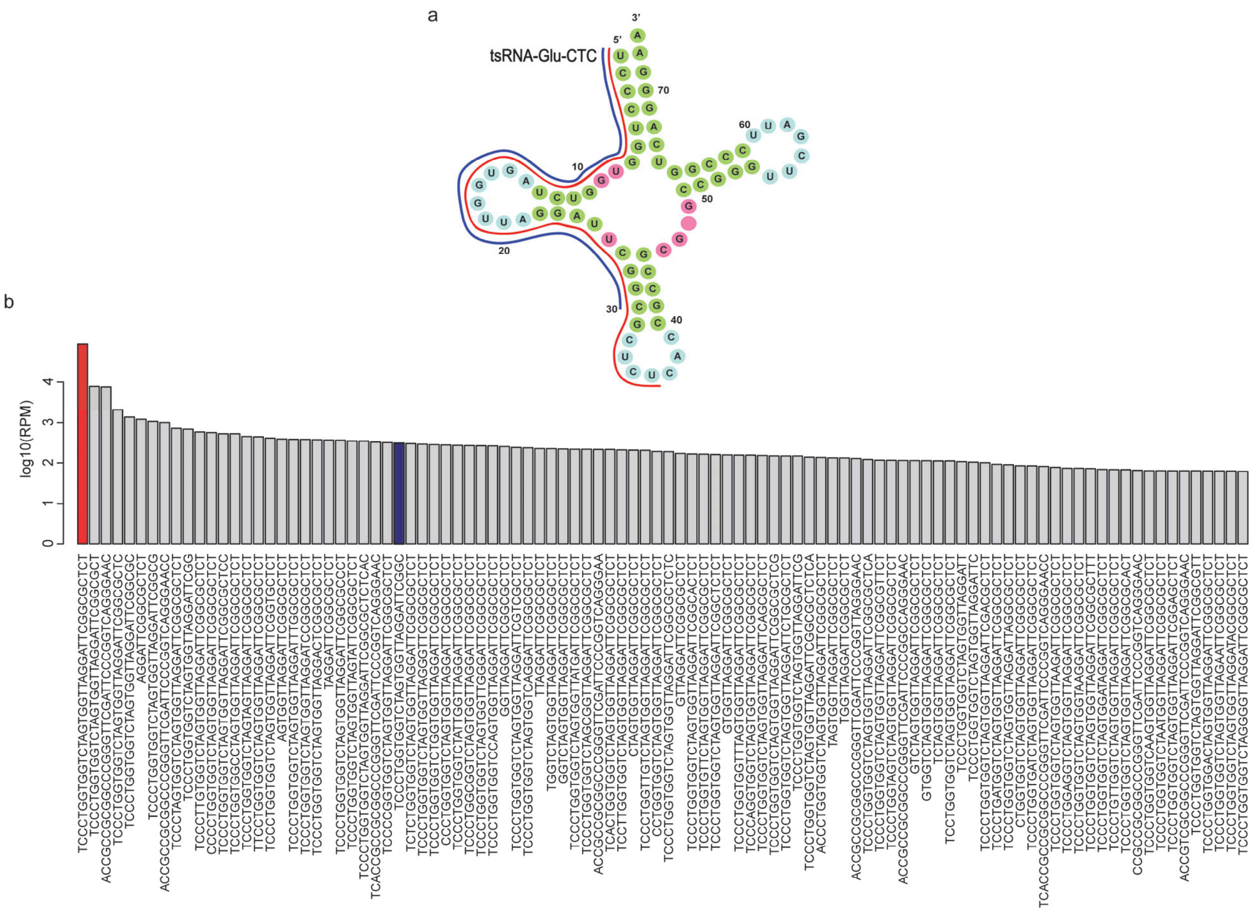

Supplementary Fig. 1. PANDORA-seq identifies different hepatic tsRNA-Glu-CTC species. Representative figure of tRNA-Glu-CTC and tsRNA-Glu-CTC (**a**) and the expression levels and sequences of different hepatic tsRNA-Glu-CTC species detected by PANDORA-seq (top 100) (**b**). The Y axis indicates read per million (RPM) in log scale (n=5 biological replicates). The two tsRNA-Glu-CTCs (35 nt and 30 nt) selected for vivo experiments are highlighted by red and blue color, respectively.

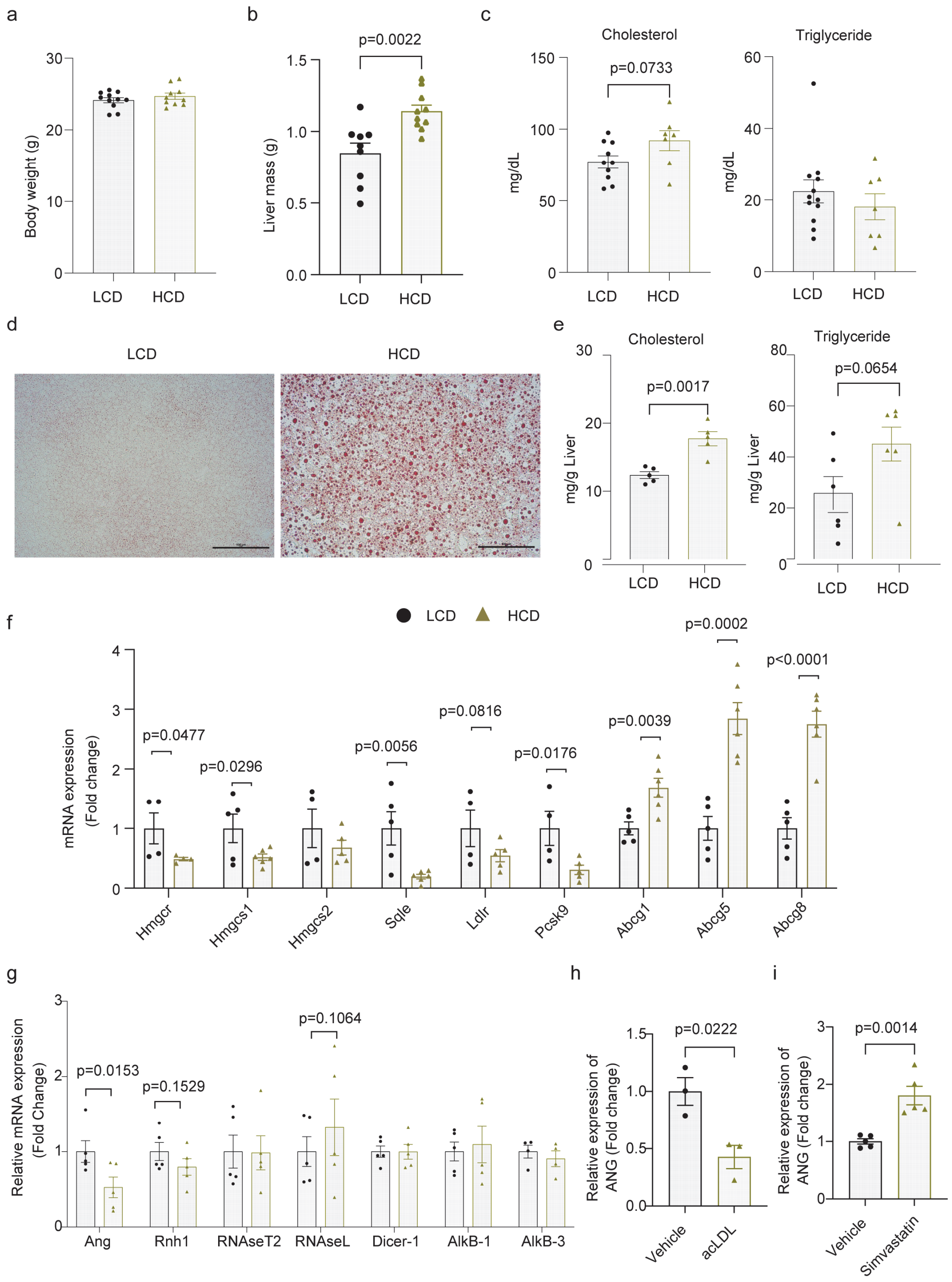

Supplementary Fig. 2. High-cholesterol diet feeding induced hepatic steatosis and altered lipogenic gene expression in wild-type mice. Eight-week-old male wide-type mice were fed a low-cholesterol diet (LCD, 0.02% cholesterol) or high-cholesterol diet (HCD, 0.5% cholesterol) for 4 weeks. Body weight (n=11,10) (**a**), liver weight (n=9,10) (**b**), serum total cholesterol (n=10,7) (left panel) and triglyceride (n=12,7) (right panel) levels (**c**), representative Oil Red-O stained liver sections (scale bar =100 μm) (**d**), and hepatic cholesterol (n=5,5) (left panel) and triglyceride (n=6,6) (right panel) contents (**e**) of LCD or HCD-fed mice. Data are shown as mean±SEM (two-tailed Student's t-test). **f**. The expression levels of hepatic lipogenic genes were analyzed by quantitative real-time PCR (n=5, mean±SEM, two-tailed Student's t test). **g**. The expression levels of indicated hepatic genes related to tsRNAs biogenesis were analyzed by quantitative real-time PCR (n=5, mean±SEM, two-tailed Student's t-test). **h** and **i**. The expression levels of ANG in human hepatic cells, HepG2 cells, treated with vehicle control, acetylated LDL (25 ug/ml) (n=3) (**h**) or simvastatin (5 uM) (n=5) (**i**) were analyzed by quantitative real-time PCR (mean±SEM, two-tailed Student's t-test). n represents the number of biological replicates.

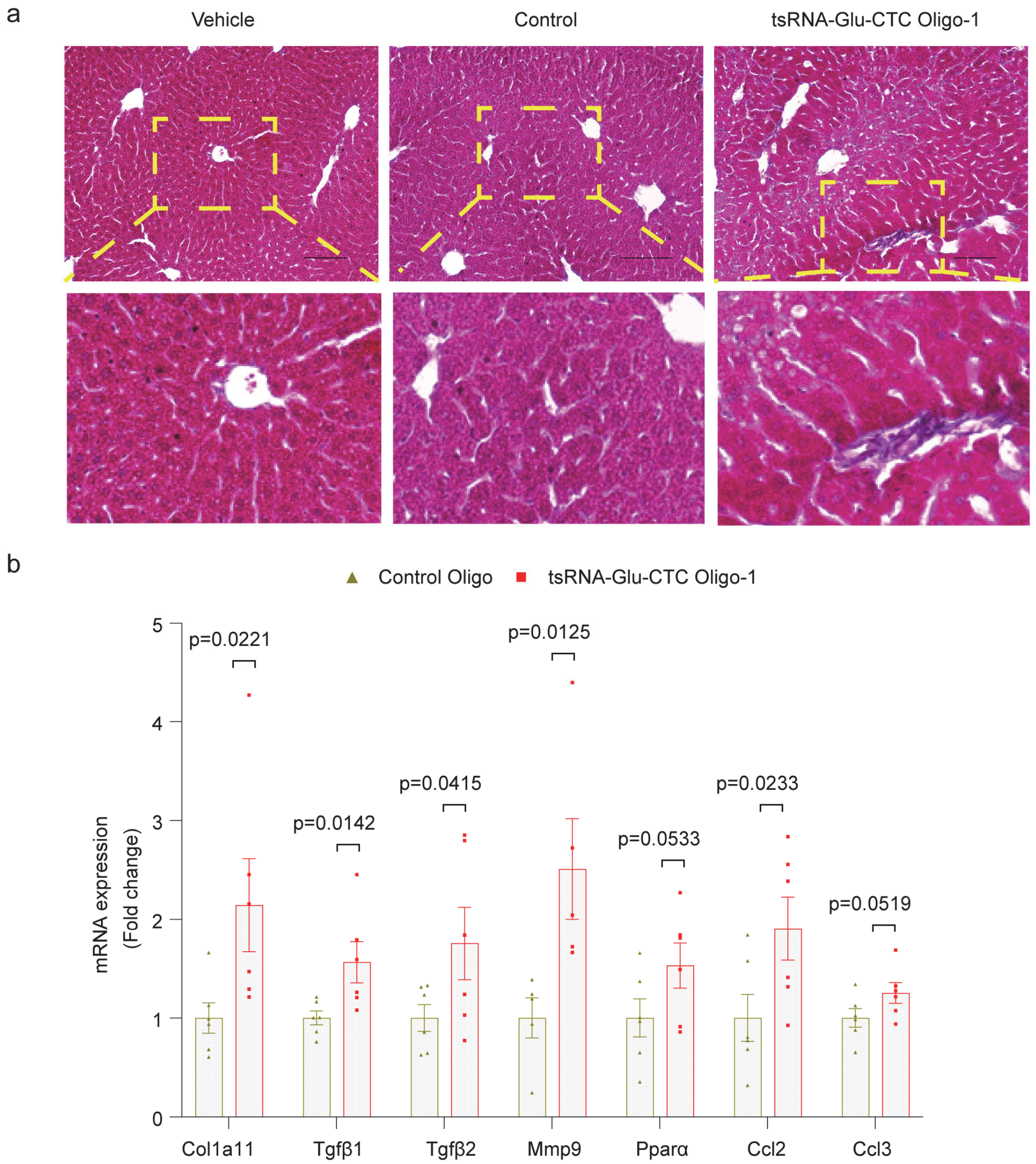

Supplementary Fig. 3. tsRNA-Glu-CTC oligonucleotide treatment affects hepatic fibrosis in mice. Eight-week-old male wild-type mice were treated with 0.6 mg/kg body weight of synthetic 30-nt tsRNA-Glu-CTC oligonucleotides, control oligonucleotides, or vehicle control once every two days for two weeks before euthanasia. **a**. Representative images of Masson's trichrome stained liver sections (scale bar =100 μm). **b**. The hepatic expression levels of fibrosis related genes were analyzed by quantitative real-time PCR (n=6, mean±SEM, two-tailed Student's t-test). n represents the number of biological replicates.

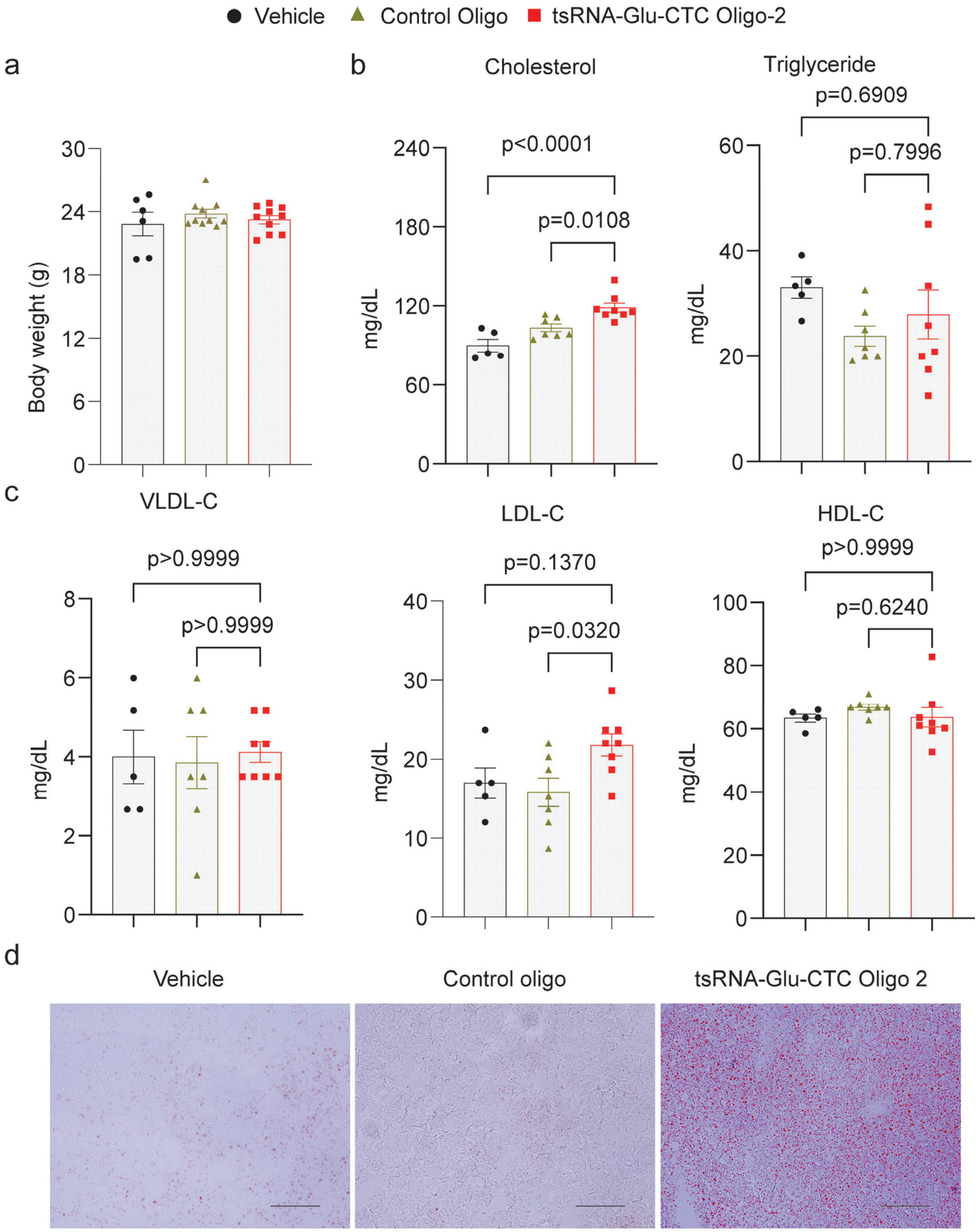

Supplementary Fig. 4. Treatment with tsRNA-Glu-CTC oligonucleotides leads to elevated circulating cholesterol levels and hepatic lipid accumulation in mice. Eight-week-old male wild-type mice were treated with vehicle control (Vehicle), 0.6 mg/kg body weight of control oligonucleotides (Control Oligo) or synthetic 35-nt tsRNA-Glu-CTC oligonucleotides (tsRNA-Glu-CTC Oligo-2) once every two days for two weeks before euthanasia. Body weight (n=6,10,10) (**a**), serum total cholesterol (left panel) and triglyceride (right panel) (n=5,7,8) (**b**), cholesterol levels of lipoprotein fractions including VLDL-C (n=5,7,7), LDL-C (n=5,7,8), and HDL-C (n=5,7,7) (**c**), and representative Oil Red-O stained liver sections (**d**) (scale bar =100 μm) of the mice. For **a-c**, data are shown as mean±SEM (one-way ANOVA, Bonferroni multiple-comparison test). n represents the number of biological replicates (mice). VLDL-C, very low-density lipoprotein cholesterol; LDL-C, low density lipoprotein cholesterol; HDL-C, high density lipoprotein cholesterol.

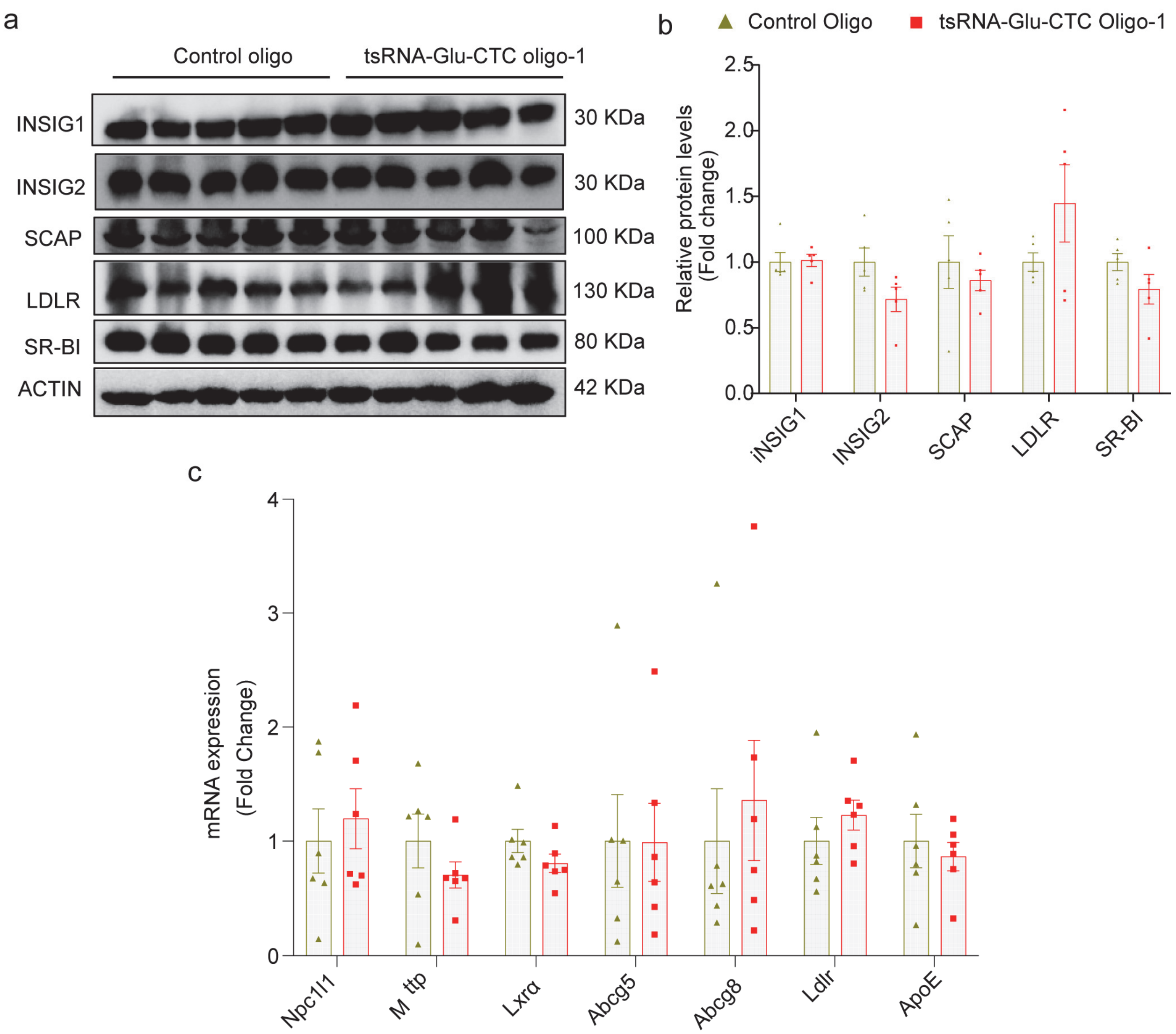

Supplementary Fig. 5. The impact of tsRNA-Glu-CTC oligonucleotide treatment on the expression levels of hepatic proteins and intestinal lipogenic genes in mice. Eight-week-old male wild-type mice were treated with 0.6 mg/kg body weight of synthetic 30-nt tsRNA-Glu-CTC oligonucleotides (tsRNA-Glu-CTC Oligo-1) or control oligonucleotides (Control Oligo) once every two days for two weeks before euthanasia. **a**. Western blot analysis of indicated proteins in the liver of mice treated with control oligonucleotides or tsRNA-Glu-CTC oligonucleotides (n=5 biological replicates). **b**. Densitometry analysis of western blot bands (normalized to Actin) (n=5 biological replicates, mean±SEM, two-tailed Student's t-test). **c**. The expression levels of lipogenic genes in the intestine were analyzed by quantitative real-time PCR (n=6 biological replicates, data are shown as mean±SEM, two-tailed Student's t-test).

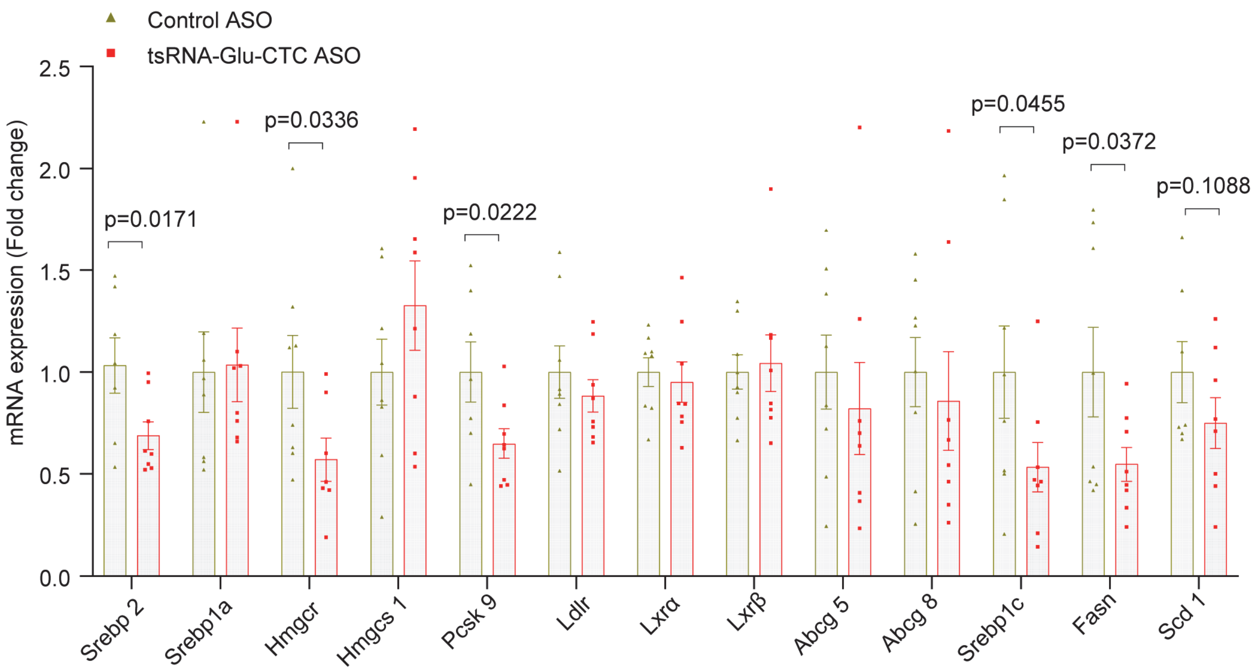

Supplementary Fig. 6. Knockdown of tsRNA-Glu-CTC by antisense oligonucleotides reduced hepatic lipogenic gene expression in mice. Eight-week-old male wild-type mice were fed a high-cholesterol diet (HCD) and were treated with tsRNA-Glu-CTC antisense oligonucleotides (ASO) or control ASO every two days for two weeks. The expression levels of hepatic lipogenic genes of mice treated with tsRNA-Glu-CTC ASO or control ASO were analyzed by quantitative real-time PCR (n=7 biological replicates for *Srebp2*, *Pcsk9*, *Scd1* in Control ASO group, n=7 biological replicates for *Hmgcs1* in tsRNA-Glu-CTC group, n=8 biological replicates for all the other data; data are shown as mean± SEM; one-tailed Student's t-test).

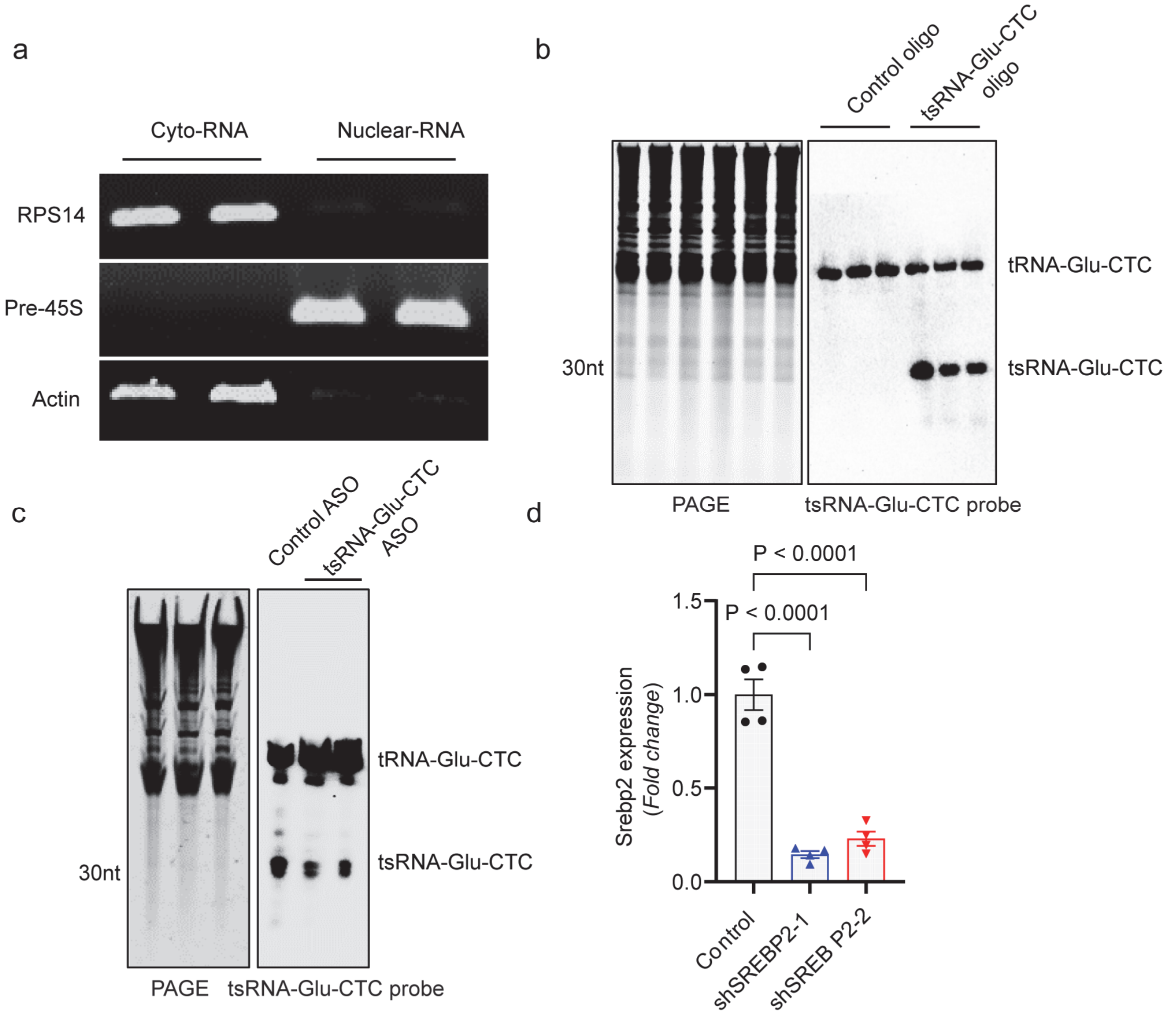

Supplementary Fig. 7. Study of tsRNA-Glu-CTC function in vitro. a. Nuclear and cytoplasmic RNAs were isolated from HepG2 cells and used for PCR analysis of cytoplasm marker RPS14, nuclear marker pre-45S and actin. Similar results were obtained in three independent experiments **b**. Northern blot analysis of tsRNA-Glu-CTC expression in HepG2 cells transfected with synthetic 30-nt tsRNA-Glu-CTC oligonucleotides or control oligonucleotides. **c**. Northern blot analysis of tsRNA-Glu-CTC expression in HepG2 cells transfected with tsRNA-Glu-CTC antisense oligonucleotides (ASO) or control ASO. Similar results were obtained in three independent experiments. **d**. HepG2 cells were transfected with control shRNAs or two different shRNAs targeting *Srebp2*. The expression levels of *Srebp2* were analyzed by quantitative real-time PCR (n=4 biological replicates, data are shown as mean ± SEM; one-way ANOVA, Bonferroni multiple-comparison test).

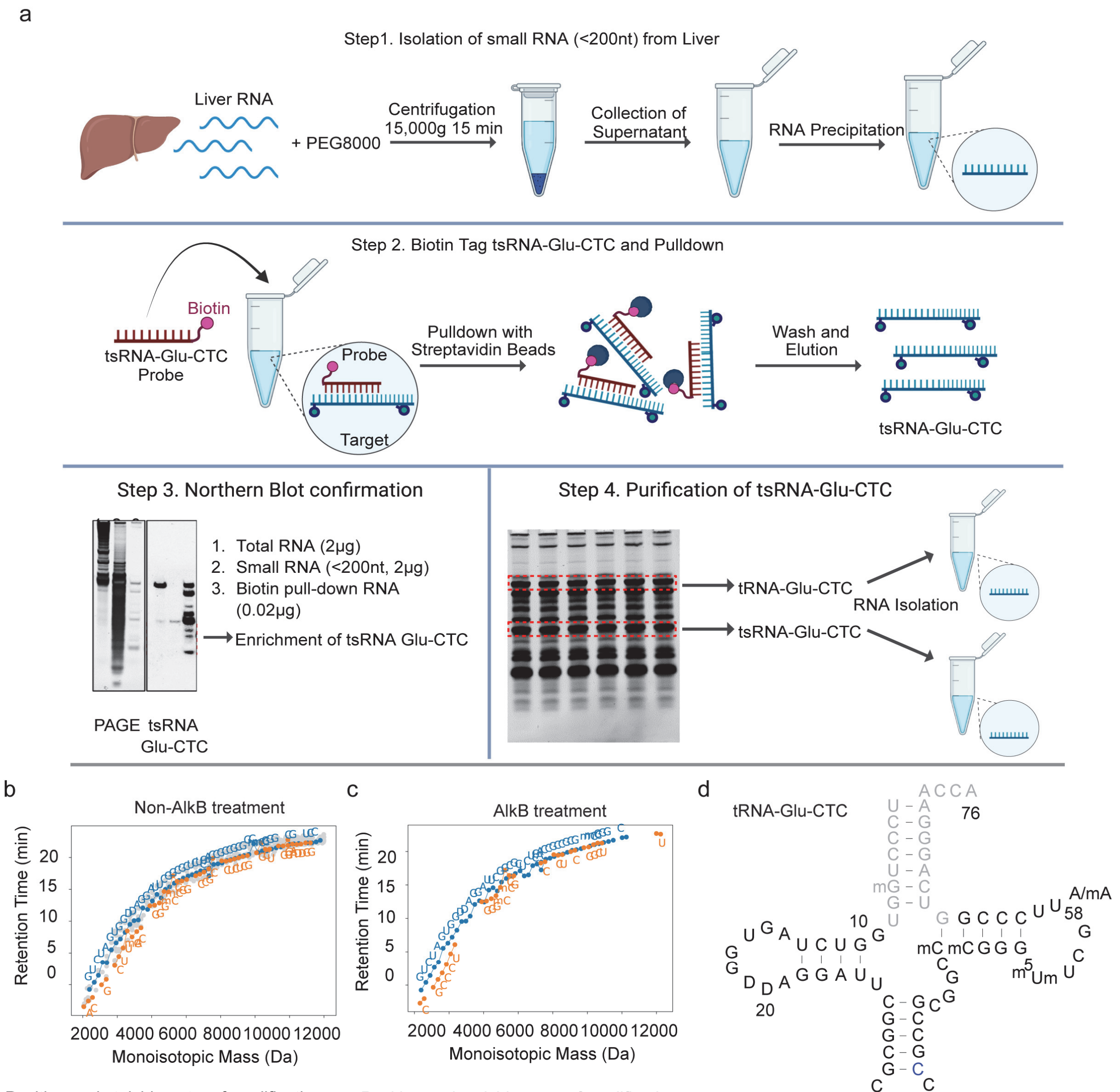

Position and stoichiometry of modifications (Non-AlkB treatment)

Postition	Base	Percentage (%)
6	Methylguanosine (mG)	100
19	Dihydrouridine (D)	100
20	Dihydrouridine (D)	100
48	5-methylcytosine (mC)	100
49	5-methylcytosine (mC)	100
53	5,2,-O--dimethyuridine	100
58	A : m1A	67:33

Position and stoichiometry of modifications (AkB treatment)

Postition	Base	Percentage (%)
6	Methylguanosine (mG)	100
19	Dihydrouridine (D)	100
20	Dihydrouridine (D)	100
48	5-methylcytosine (mC)	100
49	5-methylcytosine (mC)	100
53	5,2,-O--dimethyuridine	100
58	A : m1A	87:13

Supplementary Fig. 8. Identification of RNA modifications of endogenous tRNA-Glu-CTC by MLC-seq. a. Schematic of isolating endogenous tsRNA-Glu-CTC and mature tRNA-Glu-CTC from mouse liver. Step 1: Isolation of small RNAs (<200 nt) from hepatic total RNAs. Step 2, pull-down of endogenous tsRNA-Glu-CTC by using Biotin- labelled tsRNA-Glu-CTC probe. Step 3, Northen blot confirmation of isolated tsRNA-Glu-CTC. Step 4, Purification of tsRNA-Glu-CTC (lower band) and tRNA-Glu-CTC (upper band) from Urea PAGE gel. Created in BioRender. Zhou, C. (2025) https://BioRender.com/1zg90zg. **b-e**. MLC-seq analysis of untreated (**b**) or AlkB-treated (**c**) tRNA-Glu-CTC isolated from mouse liver. Identity, position, and stoichiometry of each modification are listed in the tables (**b** and **c**) and tRNA-Glu-CTC picture (**d**). MLC-seq results pinpoint stoichiometric changes of m^1A site-specifically at position 58 of tRNA-Glu-CTC after AlkB treatment (**e**). mG: methylguanidine; D: dihydrouridine; mC: methylcytosine; mA, methyladenine, m^5Um: 5,2'-O-dimethyluridine. MLC-seq associated data are included in Supplementary Data 1.

Supplementary Table 1. Detailed RNA-seq data of hepatic DEGs induced by tsRNA-Glu-CTC oligonucleotides in wild-type mice

Gene	log2(FC)	P-value	FC		Gene	log2(FC)	P-value	FC
Chrna4	3.702582	0.0005746	13.0193		Mphosph10	-0.694957	0.00256	0.61773
Fabp5	3.6414	3.12E-06	12.4787		Ppat	-0.696529	0.001071	0.61705
Pnpla3	3.051751	5.71E-08	8.29218		Dusp3	-0.701122	0.002648	0.61509
Nmrk2	2.913382	0.0000203	7.53382		Crem	-0.702259	0.00128	0.61461
Mvd	2.811292	7.47E-08	7.01913		Urm1	-0.704194	0.003095	0.61379
Sqle	2.810717	3.9E-10	7.01633		Trak2	-0.706235	0.001979	0.61292
Pdzk1ip1	2.735837	0.0000162	6.66145		Clk1	-0.709763	0.008384	0.61142
Ugt2b38	2.539535	0.0015687	5.81402		Fmnl2	-0.710276	0.001525	0.6112
Fdps	2.36127	0.0000337	5.13822		Ddx39a	-0.711323	0.004014	0.61076
Idi1	2.355549	7.1E-08	5.11789		B4galt1	-0.712379	0.001049	0.61031
Hmgcs1	2.218129	1.27E-06	4.6529		Tpr	-0.721921	0.00304	0.60629
Bhlhe41	2.211737	0.0000453	4.63233		Parg	-0.726747	0.008185	0.60427
Nsdhl	2.085824	0.0000088	4.24517		Ppan	-0.739723	0.000733	0.59885
Msmo1	2.041333	5.78E-07	4.11626		Rrp12	-0.740385	0.00343	0.59858
Pcsk9	2.032946	1.25E-13	4.0924		Sdc1	-0.743026	0.000672	0.59748
Acss2	2.005437	0.004151	4.0151		Gdf2	-0.743564	0.000767	0.59726
Rfx4	1.985093	0.0002439	3.95888		Reln	-0.745266	0.006906	0.59656
Gm14308	1.970226	0.0020202	3.91829		Zfp950	-0.746299	0.003668	0.59613
Acbd4	1.953051	0.0019804	3.87192		Ednra	-0.747287	0.007433	0.59572
Depp1	1.938264	0.0001188	3.83244		C9orf72	-0.748939	0.001206	0.59504
Fasn	1.937767	5.75E-06	3.83112		Tmcc3	-0.749518	0.008012	0.5948
Mycn	1.909294	0.0013371	3.75625		D16Ertd472e	-0.749592	0.000391	0.59477
Slc22a28	1.885539	0.0045522	3.69491		Gtf2e1	-0.751714	0.000933	0.5939
Cyp51	1.855803	1.22E-06	3.61953		Nol8	-0.754008	0.0074	0.59295
G6pdx	1.795554	1.05E-07	3.47149		Cc2d1b	-0.756559	0.001002	0.59191
Dclk3	1.791785	0.0092091	3.46243		Atp6v1b2	-0.77052	0.004542	0.58621
Adgrf1	1.756013	0.00267	3.37763		Zdhhc13	-0.77523	0.006363	0.5843
Pmvk	1.747679	6.88E-11	3.35818		Ndrg1	-0.77584	0.001622	0.58405
Hcn3	1.71349	0.0078928	3.27953		Ube2g2	-0.783397	0.000122	0.581
Aqp8	1.696369	0.0021499	3.24084		Slc40a1	-0.786284	0.000291	0.57984
Pklr	1.695707	0.0029914	3.23936		Skil	-0.800027	0.003394	0.57434
Me1	1.672193	8.29E-06	3.18699		Gsta4	-0.805205	0.001812	0.57228
Zfp773	1.650078	3.58E-08	3.13851		Klf2	-0.80685	0.007575	0.57163
Tbxa2r	1.618262	0.0012135	3.07005		Abcc3	-0.807046	0.000105	0.57155
Gask1a	1.617038	0.0024744	3.06745		Gldc	-0.815443	0.000786	0.56823
Bglap3	1.603531	0.0029362	3.03886		Slc25a15	-0.818834	0.001117	0.5669
Rdh11	1.596435	0.0000743	3.02395		Usp6nl	-0.82484	0.009723	0.56454
3110082I17Rik	1.589897	0.0059939	3.01028		Dop1b	-0.825798	0.006744	0.56417
Pstpip2	1.561065	0.0000063	2.95072		Mbnl2	-0.850871	0.000435	0.55445
Srgap3	1.554378	0.004416	2.93707		Kcnj8	-0.868983	0.009152	0.54753
Arrdc3	1.542581	0.0012765	2.91315		Tcf12	-0.872488	0.003846	0.5462
Fdft1	1.515987	0.0000094	2.85994		Ets2	-0.885915	0.001195	0.54114
Cenpl	1.507734	0.0000218	2.84363		Got1	-0.901817	0.002947	0.53521
Ptk6	1.503297	0.0019183	2.8349		Cth	-0.922725	0.000146	0.52751
Spatc1l	1.497185	0.0001348	2.82291		Insig2	-0.94425	1.96E-05	0.5197
Hmgcr	1.491864	0.0000446	2.81252		Arf6	-0.948178	0.002873	0.51829
Lss	1.48428	2.04E-14	2.79778		Egln3	-0.951486	0.004827	0.5171
Mapk15	1.48104	0.0057339	2.7915		Thbd	-0.953551	4.94E-05	0.51636
Egr1	1.475741	0.0002924	2.78126		Zfand2a	-0.963141	0.006428	0.51294
Dhcr7	1.441876	0.0001013	2.71674		Usp18	-0.96987	0.006406	0.51055
Mn1	1.431752	3.12E-08	2.69774		Actg1	-0.974936	0.001811	0.50876
Crygn	1.418221	0.000964	2.67256		Hexb	-0.977573	0.005886	0.50783
Fitm1	1.412232	0.0095952	2.66149		Gja1	-0.983066	0.000591	0.5059
Insig1	1.412032	8.36E-06	2.66112		Ddit4	-0.9835	0.001653	0.50575
Trpt1	1.408513	0.0019192	2.65463		Ppl	-0.984293	0.009963	0.50547
C2cd4d	1.394177	0.000981	2.62839		Nfil3	-0.993036	0.002387	0.50242
Pwwp3a	1.384648	0.0038221	2.61108		Kctd12	-0.996535	0.003118	0.5012
Nr0b2	1.384583	0.0011375	2.61096		Megf9	-1.0074	0.000313	0.49744
Rnf152	1.38258	2.17E-12	2.60734		Map3k13	-1.007696	0.007695	0.49734
Acat2	1.378102	5.36E-10	2.59926		Ung	-1.010949	0.002419	0.49622
Scd1	1.377013	0.0096235	2.5973		Desi2	-1.020816	0.00049	0.49284
Rbmxl2	1.369343	0.0021615	2.58353		Rcan2	-1.022628	0.0059	0.49222
Klhdc7a	1.358628	0.0000349	2.56441		Rab8b	-1.025114	0.006834	0.49137
Cyp2u1	1.354736	0.000089	2.5575		Acnat2	-1.028114	0.000651	0.49035
Vmn2r117	1.34852	0.0005177	2.54651		Flvcr2	-1.036646	0.006606	0.48746
Cyp8b1	1.34548	0.0000497	2.54115		Hlx	-1.052663	0.000877	0.48208
Mrnip	1.34142	0.0051113	2.53401		Clpx	-1.053895	6.46E-05	0.48167
Cfap20dc	1.340284	0.0015257	2.53201		2510009E07Rik	-1.066727	0.009086	0.4774
Lmntd2	1.310178	0.00349	2.47972		Orm2	-1.071308	0.000314	0.47589
Elovl6	1.305804	0.004113	2.47221		Ptgfrn	-1.072359	0.006389	0.47554
Eef2k	1.299917	0.0000046	2.46215		Zbtb21	-1.074654	0.00052	0.47478
Pltp	1.285138	0.0014864	2.43705		Etnppl	-1.095872	1.84E-07	0.46785
Mvk	1.276125	0.0000744	2.42188		Lbh	-1.099611	0.009514	0.46664
Dph7	1.265642	0.0096671	2.40434		Rasgef1b	-1.100823	9.08E-05	0.46625
Fads3	1.262025	0.0018817	2.39832		Spry1	-1.104208	0.003557	0.46516
Tbc1d30	1.247914	0.0008603	2.37498		Hgf	-1.107022	0.006754	0.46425
Mast3	1.244096	0.0000011	2.3687		Cyp7a1	-1.116549	0.000307	0.4612
Gabbr2	1.239691	0.0030207	2.36148		Rgs5	-1.142469	0.000575	0.45298
Fabp1	1.236669	0.0020539	2.35654		Rell1	-1.146788	0.006725	0.45163
Mid1ip1	1.233282	0.0003694	2.35101		Bloc1s6	-1.148253	0.009572	0.45117
Camk1d	1.211713	0.000154	2.31613		Gls	-1.148754	0.006301	0.45101
Cabcoco1	1.200454	0.0062635	2.29812		Fosl2	-1.156629	0.000397	0.44856
Disp2	1.171776	0.0037717	2.25289		Abca5	-1.17975	0.001031	0.44143
Dlgap1	1.154605	0.0059146	2.22623		Zmat3	-1.184281	0.001744	0.44004
Unc93a2	1.151713	0.0032369	2.22178		Nnmt	-1.192723	6.85E-05	0.43748
4931428F04Rik	1.14634	0.0030991	2.21352		Slc41a3	-1.192807	0.008729	0.43745
Erg28	1.138723	0.0001514	2.20186		Pkm	-1.209383	0.008881	0.43245
Nr1d1	1.13114	0.0029162	2.19032		Tm4sf4	-1.215968	0.001845	0.43048

Zfand4	1.128296	0.0006404	2.186		Acmsd	-1.22058	0.002999	0.42911
Paqr7	1.109442	9.28E-08	2.15762		Apold1	-1.222308	0.001018	0.4286
Sytl1	1.097309	0.000458	2.13955		Unc5b	-1.231052	0.000727	0.42601
Olfr63	1.057309	0.0021116	2.08105		Csrp1	-1.241685	0.005205	0.42288
Tlcd1	1.054807	5.11E-06	2.07744		Rab30	-1.27175	0.004275	0.41416
Hes6	1.043866	0.0056837	2.06174		Arhgef25	-1.283138	0.006776	0.4109
Mmab	1.043438	0.0001879	2.06113		Cyp4a31	-1.287653	0.004795	0.40962
Rhobtb3	1.036448	0.0003	2.05117		Adamtsl2	-1.303683	0.004584	0.40509
Cyb5r3	1.032156	0.0009821	2.04508		Kirrel	-1.310761	0.008263	0.40311
Sc5d	1.029927	0.0004531	2.04192		Myh10	-1.322527	0.00778	0.39983
Zbtb48	1.02349	0.0036038	2.03283		Saa2	-1.326466	0.007714	0.39874
Lrit1	1.014446	0.0083991	2.02013		Plk3	-1.3292	0.000466	0.39799
Zfp467	0.999203	1.29E-06	1.9989		Sik1	-1.350984	0.003948	0.39202
Il15ra	0.998701	0.0069723	1.9982		Gpx3	-1.351035	0.001827	0.39201
Vwce	0.99839	1.31E-08	1.99777		Cttnbp2nl	-1.352607	0.003027	0.39158
Mettl22	0.988322	0.0000389	1.98388		Pde1b	-1.358057	0.002025	0.39011
Sec31b	0.978537	0.0020328	1.97047		Adrb2	-1.367292	0.000285	0.38762
Tgfbr3l	0.972869	0.0018011	1.96274		Nedd9	-1.374588	0.003311	0.38566
Nxpe2	0.961239	0.0065015	1.94698		Kbtbd8	-1.396046	0.001676	0.37997
Aspg	0.953015	0.0018687	1.93591		Pde1a	-1.400998	0.002313	0.37867
Tmco6	0.952441	0.0005169	1.93514		Dpt	-1.406114	0.003067	0.37733
Hsd17b7	0.943674	0.0037522	1.92342		Map4k4	-1.408659	0.00553	0.37666
Mblac2	0.938574	0.0002423	1.91663		Rbbp8	-1.411335	9.82E-08	0.37596
Srebf1	0.929094	0.0012478	1.90408		Nt5e	-1.412897	0.000525	0.37556
Acaca	0.923799	0.0008588	1.8971		Dusp5	-1.433814	0.006739	0.37015
Shf	0.922561	5.45E-06	1.89548		Hck	-1.443162	0.009518	0.36776
Efna1	0.916176	3.36E-06	1.88711		Inhbb	-1.450385	1.69E-05	0.36592
Fzd5	0.906447	0.0000198	1.87442		Vstm4	-1.459478	0.002235	0.36362
Plaat3	0.903303	0.0097045	1.87034		Pmaip1	-1.471863	0.007338	0.36052
Tmem25	0.898786	0.0050845	1.8645		Cish	-1.488619	0.005102	0.35635
Fbxo21	0.895242	0.0029067	1.85992		1700017B05Rik	-1.490955	0.00801	0.35578
Map3k21	0.885369	0.0044761	1.84724		Cd300lf	-1.491201	0.005776	0.35572
Dixdc1	0.873463	0.0006796	1.83206		Piga	-1.496567	0.000166	0.3544
Ebpl	0.866794	0.0006129	1.82361		Osbpl5	-1.507759	0.005457	0.35166
Prpsap2	0.857837	0.0035405	1.81232		Abcb1a	-1.507935	0.00298	0.35161
Paox	0.857024	0.004683	1.8113		Prkab2	-1.512817	0.001777	0.35043
Plppr2	0.853872	0.0029753	1.80734		Lyve1	-1.516926	0.001996	0.34943
Tkt	0.84688	0.0003332	1.79861		Plekho2	-1.526674	0.000633	0.34708
Atg16l2	0.838716	0.002774	1.78846		Acot2	-1.527863	0.000316	0.34679
Mcm10	0.834439	0.0062941	1.78316		Gadd45g	-1.535261	0.001537	0.34502
Zkscan7	0.831957	0.0010945	1.7801		Abhd2	-1.540699	0.000869	0.34372
2610528J11Rik	0.821435	0.0023892	1.76716		Serpine1	-1.560183	0.007702	0.33911
Gm10033	0.811092	0.0003794	1.75454		Ccn2	-1.573973	0.004345	0.33588
Dbi	0.810791	0.0034582	1.75417		Laptm5	-1.576317	0.006593	0.33534
Thra	0.809439	0.0040639	1.75253		Ctss	-1.583765	0.009838	0.33361
Fgfr3	0.808216	0.0000831	1.75104		Cmpk2	-1.597219	0.005451	0.33051
Tecr	0.807524	0.0011052	1.75021		Ptrh1	-1.601327	0.003866	0.32957
Bhlhe40	0.801998	0.000066	1.74351		Ms4a6b	-1.612334	0.003177	0.32707
Tnfaip8l1	0.796222	0.0048099	1.73655		Sult1e1	-1.619691	0.005185	0.32541
Fam13a	0.794619	0.0021463	1.73462		Otulinl	-1.652217	0.004267	0.31815
Tmem131l	0.778503	0.0002173	1.71535		Rcan1	-1.659106	2.12E-15	0.31664
Hlcs	0.776478	0.0019008	1.71294		Tmprss2	-1.662675	0.000113	0.31585
Hsd17b2	0.775642	0.0050428	1.71195		Gm33543	-1.677461	2.14E-07	0.31263
BC003965	0.763733	0.0004745	1.69788		Ifngr1	-1.688566	8.01E-06	0.31024
Slc12a7	0.762242	0.009537	1.69612		Synpo	-1.694254	0.003871	0.30901
Gba2	0.762226	0.007482	1.69611		Cyp3a11	-1.697117	0.001308	0.3084
Abhd14a	0.762072	0.0059538	1.69592		Igfbp1	-1.705152	0.000235	0.30669
Haus7	0.759801	0.0013544	1.69326		Mdm1	-1.7248	0.005282	0.30254
Mycl	0.752535	0.0064062	1.68475		Klf6	-1.729722	0.008366	0.30151
Adrb3	0.751602	0.0001083	1.68366		Islr	-1.741998	0.007459	0.29896
Plekha6	0.749698	0.0079053	1.68144		Samd4	-1.745201	0.004268	0.29829
Rassf7	0.738425	0.0005698	1.66835		Lum	-1.761692	0.008542	0.2949
Bcar1	0.73465	0.0003082	1.66399		Robo2	-1.774664	0.003991	0.29226
Madd	0.730085	0.0000463	1.65874		Pfkp	-1.775771	0.00483	0.29204
Cpne2	0.72655	0.0097134	1.65468		Sh3bp1	-1.778635	0.005088	0.29146
Rab4a	0.726272	0.000582	1.65436		Aldh1b1	-1.782379	0.00546	0.2907
Apcs	0.720681	0.0021856	1.64796		Evi2a	-1.791058	0.006092	0.28896
Pdcd4	0.717722	0.0004687	1.64458		Coro2a	-1.794498	0.001634	0.28827
Krba1	0.717633	0.0086234	1.64448		Adamts15	-1.79608	2.95E-05	0.28796
Slc25a1	0.712427	0.0031156	1.63856		Fcgr4	-1.79926	0.005096	0.28732
Timm21	0.7114	0.0022082	1.63739		Lama2	-1.801311	0.004213	0.28691
Erbb3	0.707893	0.0038176	1.63342		Treh	-1.817778	0.00037	0.28366
Tmem220	0.702706	0.0036952	1.62755		Vcam1	-1.830358	0.009146	0.28119
Slc25a37	0.68859	0.0020375	1.61171		Tnfrsf12a	-1.833098	0.000402	0.28066
Gpam	0.683497	0.0091709	1.60603		Cpne8	-1.843718	0.00235	0.2786
1700001C19Rik	0.677313	0.0047652	1.59916		Hr	-1.861794	0.002627	0.27513
Scap	0.675155	0.0010998	1.59677		Abcc4	-1.884282	0.004482	0.27088
Prxl2a	0.659534	0.0016137	1.57957		Plaur	-1.88817	0.005279	0.27015
Tmie	0.658793	0.0017213	1.57876		Engase	-1.902746	0.000476	0.26743
Srebf2	0.658729	0.0074325	1.57869		Myc	-1.948723	0.009324	0.25905
Thnsl2	0.654141	0.0001401	1.57368		Pla2g7	-1.94959	0.001041	0.25889
Abcc10	0.642182	0.0015216	1.56069		Gsta2	-1.959685	0.000875	0.25708
Cdc14b	0.64051	0.0030654	1.55888		Evl	-1.961148	0.009787	0.25682
Anks4b	0.635003	0.0034658	1.55294		Gpat3	-1.964559	9.74E-06	0.25622
Gorasp1	0.634198	0.0034298	1.55207		Cidec	-1.967229	0.003426	0.25574
Dnase1l3	0.632865	0.003877	1.55064		Atp8b5	-1.973879	0.004098	0.25457
Cfap20	0.632435	0.0024637	1.55018		Oas3	-2.00968	0.003788	0.24833
Tmem254a	0.616443	0.0063259	1.53309		Ltbp1	-2.012031	0.008881	0.24792
Hjurp	0.611875	0.0025284	1.52824		Arhgap11a	-2.019364	0.009942	0.24667
Entpd8	0.61031	0.0093897	1.52659		Plcg2	-2.04742	0.00096	0.24192
Zmym3	0.608524	0.0061811	1.5247		Cerk	-2.060706	0.006473	0.2397
Prkcz	0.605383	0.0014318	1.52138		Cxcl10	-2.065041	0.006636	0.23898
Rara	0.604376	0.0011813	1.52032		Fgd1	-2.074464	0.008007	0.23742
Ino80b	0.600438	0.0029868	1.51618		Cd300c2	-2.088872	0.004396	0.23506

Cxxc5	0.598064	0.0011188	1.51368		Rasd1	-2.099999	4.26E-05	0.23326
Stard13	0.596724	0.0009565	1.51228		Slc16a5	-2.122964	2.55E-06	0.22957
Pcyt2	0.596101	0.0069029	1.51163		Hmmr	-2.137743	0.004918	0.22724
Tecpr1	0.59473	0.0035643	1.51019		Arntl	-2.150336	0.003269	0.22526
Pgrmc2	0.578698	0.0065859	1.4935		Phlda3	-2.155785	0.008067	0.22441
Clec4g	0.575881	0.0018364	1.49059		Tlr7	-2.215682	0.003236	0.21528
Cbx4	0.575747	0.0017817	1.49045		Tes	-2.216436	0.002755	0.21517
Elmo3	0.573009	0.0015721	1.48762		Pls1	-2.221199	0.002187	0.21446
Prkra	0.568236	0.0051026	1.48271		Plek	-2.221593	0.009797	0.2144
Tmem184a	0.559343	0.0099868	1.4736		Stmn1	-2.222447	0.001695	0.21428
Sptbn2	0.557608	0.0080955	1.47183		Grhl1	-2.230348	5.41E-06	0.21311
Prr12	0.557146	0.0029882	1.47136		Cyp3a59	-2.243648	5.05E-05	0.21115
Pde4c	0.552798	0.0037772	1.46693		Itgb2	-2.26408	0.002727	0.20818
Osgep	0.551829	0.0051771	1.46594		Pdk4	-2.266086	8.11E-05	0.20789
Nfyb	0.550993	0.0009431	1.46509		Nr4a1	-2.273453	0.000245	0.20683
Dnajc4	0.539756	0.0086198	1.45373		Nnat	-2.274702	7.95E-06	0.20666
Ehd3	0.536609	0.0070122	1.45056		Ccl6	-2.282848	0.000818	0.20549
Capn10	0.535563	0.0049611	1.44951		Clec12a	-2.285527	0.008277	0.20511
Fam20c	0.534534	0.0073436	1.44847		Ntrk2	-2.287509	0.005576	0.20483
Slc25a45	0.533693	0.0067235	1.44763		Nusap1	-2.309255	7.76E-05	0.20176
Hykk	0.53179	0.0082173	1.44572		Slc1a4	-2.329384	0.006816	0.19897
Selenok	0.522943	0.0065747	1.43688		Smc4	-2.352073	0.000547	0.19586
Gpr146	0.520826	0.0041541	1.43478		Hapln1	-2.357724	0.000131	0.1951
Vegfb	0.515172	0.0037778	1.42916		Hoxa5	-2.360779	0.002156	0.19469
Gpr182	0.505185	0.007864	1.41931		Vav1	-2.381065	0.001692	0.19197
Hook2	0.501572	0.0032393	1.41576		Trp53inp1	-2.383393	4.98E-05	0.19166
Dhrs3	0.497121	0.0088251	1.41139		Mt1	-2.394343	0.001256	0.19021
Nelfe	0.488217	0.0053411	1.40271		Rsad2	-2.418106	1.99E-05	0.1871
Rufy3	0.467773	0.0094556	1.38297		Krt23	-2.422955	0.003179	0.18647
Cgn	0.446818	0.0073747	1.36303		Mmp2	-2.437061	0.0083	0.18466
Abcb8	0.44147	0.008119	1.35799		Elovl7	-2.440085	0.007737	0.18427
Samd4b	-0.48047	0.0078755	0.71674		Cxcr4	-2.569691	0.002542	0.16844
Ablim3	-0.48613	0.0078501	0.71394		Lcn2	-2.580255	2.14E-06	0.16721
G3bp2	-0.49345	0.0043457	0.71032		Csf2rb2	-2.59274	0.000635	0.16577
Atxn7l3	-0.50758	0.0072433	0.7034		Mthfd1l	-2.594373	0.001486	0.16558
Pde12	-0.50969	0.0062179	0.70237		Osbpl3	-2.635996	0.001491	0.16087
Rlim	-0.5264	0.0013846	0.69428		Ltb	-2.716222	0.000239	0.15217
Cybc1	-0.53706	0.0095419	0.68917		Moap1	-2.746108	0.001223	0.14905
Antxr2	-0.54297	0.0030719	0.68636		Ifi27l2b	-2.765324	0.0031	0.14708
Ptk2	-0.54832	0.0075428	0.68382		Lpl	-2.835649	0.006531	0.14008
Ago2	-0.56176	0.0023115	0.67748		Lgals3	-2.851309	0.001365	0.13857
Rrs1	-0.58526	0.0092663	0.66653		Rbak	-2.931508	0.006573	0.13108
Ikbkg	-0.58858	0.0083654	0.665		Cbr3	-2.949286	0.001693	0.12947
Traf2	-0.58939	0.0022284	0.66462		Cdkn1a	-2.975762	5.68E-05	0.12712
Lancl1	-0.59169	0.0077569	0.66356		Cip2a	-3.015245	0.002284	0.12369
Dst	-0.59756	0.0037161	0.66087		Mt2	-3.075535	0.000608	0.11862
Lrrc58	-0.61302	0.0018068	0.65383		Cyp2b10	-3.092059	3.28E-05	0.11727
Ermp1	-0.6148	0.0039719	0.65302		Sh3pxd2b	-3.108791	0.004513	0.11592
Mrtfb	-0.62232	0.0026181	0.64962		Cyp4f16	-3.123643	0.005183	0.11473
Sdsl	-0.62432	0.0094341	0.64873		Col1a1	-3.147372	0.006987	0.11286
Lrrc8a	-0.62652	0.0002271	0.64774		Cdca3	-3.155545	0.002255	0.11222
Tnrc6c	-0.63286	0.005618	0.6449		Ecm2	-3.528569	9.87E-07	0.08666
Steap4	-0.63877	0.0042934	0.64226		Arg2	-3.675713	0.000632	0.07825
Pprc1	-0.64303	0.0036461	0.64037		H1f10	-3.737333	0.000418	0.07498
Cpne1	-0.65444	0.0068265	0.63532		Lysmd2	-3.768114	0.000286	0.0734
**Gene**	**log2(FC)**	**P-value**	**FC**		**Gene**	**log2(FC)**	**P-value**	**FC**
Prpf38a	-0.65706	0.0098739	0.63417		Dusp8	-3.933711	0.000243	0.06544
Apol9a	-0.66243	0.0078101	0.63181		Atf3	-4.127837	2.58E-05	0.0572
Mapkapk2	-0.66322	0.0007999	0.63147		Primpol	-4.912069	0.00015	0.03321
Kpna2	-0.66454	0.0026452	0.63089		Lrtm2	-5.696548	0.000171	0.01928
Mfap3	-0.68198	0.005465	0.62331		Cyp2a4	-6.90242	0.001535	0.00836
Noct	-0.69381	0.0034725	0.61822		Fam177a	-9.437567	3.37E-09	0.00144

The edgeR tool was applied to identify DEGs with P-value <0.01 and a fold change (FC) >1.5 as a cut-off threshold (n=4 mice for control oligo, n=3 mice for tsRNA-Glu-CTC oligo).

Supplementary Table 2. Sequences of RNA oligonucleotides and Northern blot probes

	Sequence
Oligo Control-1	5’rUrArCrGrGrArCrUrUrArArGrCrGrGrCrUrArCrArUrArUrUrGrCrArCrUrU-3’
Oligo Control-2	5'rUrGrCrArUrCrGrArGrArGrGrCrArCrUrCrGrUrArCrGrUrArUrGrArGrArCrUrCrArCrG-3'
tsRNA-Glu-CTC Oligo-1	5’rUrCrCrCrUrGrGrUrGrGrUrCrUrArGrUrGrGrUrUrArGrGrArUrUrCrGrGrC-3’
tsRNA-Glu-CTC Oligo-2	5’rUrCrCrCrUrGrGrUrGrGrUrCrUrArGrUrGrGrUrUrArGrGrArUrUrCrGrGrCrGrCrUrCrU-3’
miRNA-122 Oligo	5’rUrGrGrArGrUrGrUrGrArCrArArUrGrGrUrGrUrUrU-3’
ASO-Control	5’+T*+A*+C*+G*C*A*C*T*A*G*T*G*G*T*C*+C*+A*+G*+G-3’
ASO-tsRNA-Glu-CTC	5'+T*+A*+A*+C*C*A*C*T*A*G*A*C*C*A*C*+C*+A*+G*+G-3'
Control RNA probe-Biotin	5’Biosg/TACGCACTAGTGGTCCAGG-3'
tsRNA-Glu-CTC probe-Biotin	5’Biosg/TAACCACTAGACCACCAGG-3'
Control RNA-Biotin-PD	5’rUrArCrGrGrArCrUrUrArArGrCrGrGrCrUrArCrArUrArUrUrGrCrArCrUrU-Biosg/3’
tsRNA-Glu-CTC-Biotin-PD	5’rUrCrCrCrUrGrGrUrGrGrUrCrUrArGrUrGrGrUrUrArGrGrArUrUrCrGrGrC-Biosg/3’
tsRNA-Glu-CTC NB probe	5'Dig-TAACCACTAGACCACCAGG-3'
tsRNA-Ser-GCT NB probe	5'Dig-CGAACCCACGCGTGCAGAG-3’
miRNA-122 NB probe	5'Dig-AAACACCATTGTCACACTCCA-3'

*: Phosphorothioate bonds; +: Affinity Plus

Supplementary Table 3. Primer Sequences used for QPCR and PCR

Genes	Primer sequences	Genes	Primer sequences
mActin	5'-GGCTGTATTCCCCTCCATCG-3'	mApoB	5'-ATGGGAAGAAACAGGCTTGA-3'
	5'-CCAGTTGGTAACAATGCCATGT-3'		5'-TTCTGTCCCACGAATTGACA-3'
mHMGCR	5'-AGCTTGCCCGAATTGTATGTG-3'	mPCSK9	5'-TTGCAGCAGCTGGGAACTT-3'
	5'-TCTGTTGTGAACCATGTGACTTC-3'		5'-CCGACTGTGATGACCTCTGGA-3'
mHMGCS1	5'-CCTTCAGGGGTCTAAAGCTGGAAG-3'	mABCG8	5'-GTAGCTGATGCCGATGACAA-3'
	5'-CAGCCAATTCTTGGGCAGAGTG-3'		5'-GGGGCTGATGCAGATTCA-3'
mSREBP2	5'-GCGTTCTGGAGACCATGGA-3'	mFASN	5'-GGAGGTGGTGATAGCCGGTAT-3'
	5'-ACAAAGTTGCTCTGAAAACAAATCA-3'		5'-TGGGTAATCCATAGAGCCCAG-3'
mSREBP1a	5'-GCGCCATGGACGAGCTG-3'	mSCD1	5'-TTCTTGCGATACACTCTGGTGC-3'
	5'-TTGGCACCTGGGCTGCT-3'		5'-CGGGATTGAATGTTCTTGTCGT-3'
mSREBP1c	5'-GGAGCCATGGATTGCACATT-3'	mCCL13	5'-CCTGGCTCTTGCTTGCCTT-3’
	5'-GCTTCCAGAGAGGAGGCCAG-3'		5'-GGTCTTGTGTGATGTTGCTCA-3’
mLXRα	5'-AGGAGTGTCGACTTCGCAAA-3'	mANG	5'-CATCCCAACAGGAAGGAAGGA-3’
	5'-CTCTTCTTGCCGCTTCAGTTT-3'		5'-ACCTGGAGTCATCCTGAGCC-3’
mLXRβ	5'-AAGCAGGTGCCAGGGTTCT-3'	mRNH1	5'-TCCAGTGTGAGCAGCTGAGT-3’
	5'-TGCATTCTGTCTCGTGGTTGT-3'		5'-TGAGCTGATGTCTTTGCACC-3’
mHMGCS2	5'-GAAGAGAGCGATGCAGGAAAC-3'	mTGFb1	5'-CCACCTGCAAGACCATCGAC-3’
	5'-GTCCACATATTGGGCTGGAAA-3'		5'-CTGGCGAGCCTTAGTTTGGAC-3’
mLDLR	5'-GAAGTCGACACTGTACTGACCACC-3'	mTGFb2	5'-TCCCCTCCGAAAATGCCATC-3’
	5'-CTCCTCATTCCCTCTGCCAGCCAT-3'		5'-TGAGACATCAAAGCGGACGA-3’
mABCG5	5'-TGCCCATTCCTTTAAAAATCC -3'	mCol1A1	5'-CACCTGGTCCACAAGGTTTC-3’
	5'-GATGAACTGGACCCCTTGG -3'		5'-ATCTCCATTCTTGCCAGGAG-3’
mDHCR7	5'-AGCACAACGCTCCCAAAGT-3'	mRNAaseT2	5'-ACTATGGCCCGATAGAGCAGA-3’
	5'-GCCCCATTGTCCTTGAGAT-3’		5'-ATTGGCTGCGATTAGAAGACC-3’
mSQLE	5'-ATAAGAAATGCGGGGATGTCAC-3’	mRNAaseL	5'-TAGGCGAACACATCAATGAGGA-3’
	5'-ATATCCGAGAAGGCAGCGAAC-3’		5'-CTGCCTCTGGAACGCTGAG-3’
mABCG1	5'-AGGTCTCAGCCTTCTAAAGTTCCTC-3’	mDier-1	5'-GGTCCTTTCTTTGGACTGCCA-3’
	5'-TCTCTCGAATGAAATTTATCG-3’		5'-GCGATGAACGTCTTCCCTGA-3’
mSRBI	5'-CTCATCAAGCAGCAGGTGCTCA-3’	mALKB1	5'-AAGCGAAGACCCCGAAGTTTA-3’
	5'-GAGGATTCGGGTGTCATGAA-3’		5'-CAGTGGCGACTTGCTCTGA-3’
mCCL2	5'-TTAAAAACCTGGATCGGAACCAA-3’	mALKB3	5'-GCCAGGTAGCCATCCCTT-3’
	5'-GCATTAGCTTCAGATTTACGGGT-3’		5'-AGGGGAAGCTGGCTGAGT-3’
mNPC1L1	5'-TTGCCTTGACCTCTGGCTTAG-3’	hSREBP2	5'-GCTGAAGCTGGCAAATCA AAAGAAC-3’
	5'-AGGGCGGATGAATCTGTGC-3’		5'-TCATCCAATAGAGGGCTTCCTGGCT-3’
mApoE	5'-ATTGCTGACAGGATGCCTAGC-3’	hRPS14	5’-GGCAGACCGAGATGAATCCTC -3’
	5'-GGTTGGTTGCTTTGCCACTC-3’		5’-CAGGTCCAGGGGTCTTGGTCC -3’
mMTTP	5'-TGAGCGGCTATACAAGCTCAC-3’	hPre-45S	5’-CCGCGCTCTACCTTACCTAC -3’
	5'-CTGGAAGATGCTCTTCTCGC-3’		5’-GAGCGACCAAAGGAACCATA -3’
mInsig1	5'-TCACAGTGACTGAGCTTCAGCA-3’	hActin	5’-CATGTTTGAGACCTTCAACAC -3’
	5'-TCATCTTCATCACACCCAGGAC-3’		5’-CCAGGAAGGAAGGCTGGAA -3’
mInsig2	5'-CCCTCAATGAATGTACTGAAGGATT-3’	hSREBP2-Promoter	5'-GAGAAGCGGGACTTGGATG -3'
	5'-TGTGAAGTGAAGCAGACCAATGT-3’		5'-GCCTTCTCCGTTGGATCTG -3'
mMMP9	5'-CTGGACAGCCAGACACTAAAG-3’	hMTTP-Promoter	5’-CATCCAGCCTGTTTGGGAACTCAG-3’
	5'-CTCGCGGCAAGTCTTCAGAG-3’		5’-GTGGGAGGGTAGTAAGGATTCTC-3’
mPPARa	5'-TGTCGAATATGTGGGGACAA-3’	hCYP3A4-Promoter	5’-ATGCCAATGGCTCCACTTGAG -3’
	5'-AATCTTGCAGCTCCGATCAC-3’		5’-CTGGAGCTGCAGCCAGTAGCAG -3’

Supplementary Table 4. Primer sequences used for *Srebp2* promoter reporter construction

	Primers sequence
Truncated plasmids Reverse primer	5'-CTTGATATCTTTGAGCTGCGCCCCGACT-3'
p793-Luc Forward primer	5'-CGGGGTACCGGGGGCGGTGGTAGAAGGTAG-3'
p485-Luc Forward primer	5'-CGGGGTACCGGGCCAGTTGTAAAATGTTCAC-3'
p300-Luci Forward primer	5'-CGGGGTACCTGCCCTGATATTCTACTGTTTGGA-3'
p245-Luci Forward primer	5'-CGGGGTACCGCTGCAATACCTTCACTGGAGAC-3'
E-Box Mutation-Forward primer	5'-GGGGG AGGGAAGAGGCCCAAGGGGAGATGACGTAATGTGC-3'
E-BoX Mutation-Reverse primer	5'-TACGTCATCTCCCCTTGGGCCTCTTCCCTCCCCCTAGCTC-3'
