# Supplementary material for: A cholesterol-responsive hepatic tRNA-derived small RNA regulates cholesterol homeostasis and atherosclerosis development
Source: Nat Commun. 2025 Dec 15;16:11043. doi: 10.1038/s41467-025-67387-z (PMC12706008; doi:10.1038/s41467-025-67387-z)
Supplement: Supplementary file 2 — Description Of Additional Supplementary File [file 41467_2025_67387_MOESM2_ESM.pdf]

Description of Additional supplementary files

Supplementary Data 1: Mass spectrometry (MS) data for all ladder fragments derived from the isolated endogenous tsRNA-Glu-CTC and tRNA-Glu-CTC (Non-AlkB treatment and AlkB treatment). The data are associated with Fig 9a-9c and Supplementary Fig. 8b-8e.
