# Supplementary material for: A cholesterol-responsive hepatic tRNA-derived small RNA regulates cholesterol homeostasis and atherosclerosis development
Source: Nat Commun. 2025 Dec 15;16:11043. doi: 10.1038/s41467-025-67387-z (PMC12706008; doi:10.1038/s41467-025-67387-z)
Supplement: Supplementary file 4 — Reporting Summary [file 41467_2025_67387_MOESM4_ESM.pdf]

nature portfolio

Corresponding author(s): Changcheng Zhou

Last updated by author(s): Oct 24, 2025

Reporting Summary

Nature Portfolio wishes to improve the reproducibility of the work that we publish. This form provides structure for consistency and transparency in reporting. For further information on Nature Portfolio policies, see our Editorial Policies and the Editorial Policy Checklist.

Statistics

For all statistical analyses, confirm that the following items are present in the figure legend, table legend, main text, or Methods section.

n/a	Confirmed	
☐	☒	The exact sample size (*n*) for each experimental group/condition, given as a discrete number and unit of measurement
☐	☒	A statement on whether measurements were taken from distinct samples or whether the same sample was measured repeatedly
☐	☒	The statistical test(s) used AND whether they are one- or two-sided<br>*Only common tests should be described solely by name; describe more complex techniques in the Methods section.*
☐	☒	A description of all covariates tested
☐	☒	A description of any assumptions or corrections, such as tests of normality and adjustment for multiple comparisons
☐	☒	A full description of the statistical parameters including central tendency (e.g. means) or other basic estimates (e.g. regression coefficient) AND variation (e.g. standard deviation) or associated estimates of uncertainty (e.g. confidence intervals)
☐	☒	For null hypothesis testing, the test statistic (e.g. *F*, *t*, *r*) with confidence intervals, effect sizes, degrees of freedom and *P* value noted<br>*Give P values as exact values whenever suitable.*
☒	☐	For Bayesian analysis, information on the choice of priors and Markov chain Monte Carlo settings
☒	☐	For hierarchical and complex designs, identification of the appropriate level for tests and full reporting of outcomes
☐	☒	Estimates of effect sizes (e.g. Cohen's *d*, Pearson's *r*), indicating how they were calculated

Our web collection on statistics for biologists contains articles on many of the points above.

Software and code

Policy information about availability of computer code

Data collection	The Pandora-seq small RNA sequence data were obtained by UCSD IGM Genomics Center.<br>Immunofluorescence mages were captured on a Nikon Eclipse Ti2 light microscope (Nikon) Zeiss LSM 880 Confocal Laser Scanning Mircoscope.<br>QPCR data were obtained on CFX96TM real-time System (Biorad).<br>Luminescence data were acquired by Synergy h1 Microplate Reader (BioTek).<br>Western blotting and Northern blotting images data were collected by ChemiDoc imaging System.
Data analysis	Small RNA sequence data were annotated using the software SPORTS1.1 (V1.1.0). MLC-seq data were processed using Therma BioPharma Finder 4.0. Statistical analyses were conducted by GraphPad prism (V10.0.1).

For manuscripts utilizing custom algorithms or software that are central to the research but not yet described in published literature, software must be made available to editors and reviewers. We strongly encourage code deposition in a community repository (e.g. GitHub). See the Nature Portfolio guidelines for submitting code & software for further information.

Data

Policy information about availability of data

All manuscripts must include a data availability statement. This statement should provide the following information, where applicable:

- Accession codes, unique identifiers, or web links for publicly available datasets
- A description of any restrictions on data availability
- For clinical datasets or third party data, please ensure that the statement adheres to our policy

Bulk RNA sequence, PANDORA-seq, and small RNA-seq data have been deposited in the Gene Expression Ommibus (GSE300043). Values for graphs in the figures and supplemental figures are provided in the Source Data file with this manuscript.

Research involving human participants, their data, or biological material

Policy information about studies with human participants or human data. See also policy information about sex, gender (identity/presentation), and sexual orientation and race, ethnicity and racism.

Reporting on sex and gender	This study included 7 men (41%) and 10 women (59%).
Reporting on race, ethnicity, or other socially relevant groupings	Self-reported race categories include Hispanic/Latino (N=7), Asian (N=5), White (N=3), and Black (N=2)
Population characteristics	We recruited healthy men and women over the age of 18 with no current COVID-19 infection or pregnancy. Individuals who were currently incarcerated or were not residents of Riverside County, CA, were excluded. The mean age was 25.14± 1.28 years old for men and 34.70 ± 4.27 years old for women, and body mass index was 29.94 ± 2.54 kg/m2 for men and 26.36 ± 1.58 kg/m2 for women.
Recruitment	Participants were recruited from Riverside Free Clinic, University of California Riverside Health Clinic, and University of California, Riverside through word of mouth and advertising.
Ethics oversight	All human subjects research was approved by the UCR IRB (Protocol #HS-20-128).

Note that full information on the approval of the study protocol must also be provided in the manuscript.

Field-specific reporting

Please select the one below that is the best fit for your research. If you are not sure, read the appropriate sections before making your selection.

☒ Life sciences ☐ Behavioural & social sciences ☐ Ecological, evolutionary & environmental sciences

For a reference copy of the document with all sections, see nature.com/documents/nr-reporting-summary-flat.pdf

Life sciences study design

All studies must disclose on these points even when the disclosure is negative.

Sample size	Experimental sample sizes are included in the figure legends. The minimal sample size was decided based on power analysis of samples of our previous publications, preliminary data, and established protocols to ensure a statistical power at the level (1-β) of 80% and a significance level (α) of 5% for t-test.
Data exclusions	No data were excluded.
Replication	All experiments were performed in at least 3 biological replicates, which is indicated in the Figure Legends.
Randomization	Cells and animals were randomly assigned to experimental conditions.
Blinding	Sample collection is not blinded. Samples for small RNA-seq were sent out in single-blinded manner. Blinding were applied during data analyses.

Reporting for specific materials, systems and methods

We require information from authors about some types of materials, experimental systems and methods used in many studies. Here, indicate whether each material, system or method listed is relevant to your study. If you are not sure if a list item applies to your research, read the appropriate section before selecting a response.

Materials & experimental systems

n/a	Involved in the study
☐	☒ Antibodies
☐	☒ Eukaryotic cell lines
☒	☐ Palaeontology and archaeology
☐	☒ Animals and other organisms
☐	☒ Clinical data
☒	☐ Dual use research of concern
☒	☐ Plants

Methods

n/a	Involved in the study
☒	☐ ChIP-seq
☒	☐ Flow cytometry
☒	☐ MRI-based neuroimaging

Antibodies

Antibodies used

Northern blotting: Anti-Digoxigen-AP Fab fragments (Roach, REF:11093274910) used in Fig. 1g, h and j, Fig. 2c, d, e and f, Fig. 3b, Fig. 4b, Fig. 6a, Fig. 7b and c, Fig. 8a and c are diluted in 1: 10000.
Western blotting: Anti-HMGCR (NovusBio, Cat No. NBP2-66888) used in Fig. 5f and Fig. 8k are diluted in 1:1000. Anti-SREBP2 (ProteinTech, Cat No. 28212-1-AP) used in Fig. 5f and Fig. 9k. are diluted in 1:1000.
Anti-PSCK9 (Abcam, Cat No. ab28770) used in Fig. 5f and Fig. 9k. are diluted in 1:1000. Anti-GAPDH (Sigma, Cat No. G9545) used in Fig. 5f and Fig. 9k. are diluted in 1:2000. Anti-Insig1 (ProteinTech, Cat No. 55282-1-AP) used in Suplementary Fig. 5a. are diluted in 1:1000. Anti-Insig2 (ProteinTech, Cat No. 24766-1-AP) used in Supplementary Fig. 5a. are diluted in 1:1000. Anti-Scap (ThermoFisher, Cat No. PA5-2898) used in Supplementary Fig. 5a. are diluted in 1:1000. Anti-LDLR (Abcam, Cat No. ab52818) used in Suplementary Fig. 5a. are diluted in 1:1000. Anti-SR-BI (NovusBio, Cat No. NB400-104) used in Suplementary Fig. 5a. are diluted in 1:1000. Anti-Actin (Sigma, Cat No. A2066) used in Supplementary Fig. 5a. are diluted in 1:1000.
Immunofluorescence: Anti-CD68 (Bio-Rad AbD Serotec; catalog no.: MCA1957) used in Fig. 7j are diluted in 1:50.
CHIP: Anti-SREBP2 (Abcam, Cat No. ab30682) used in Fig. 6i, 4ug antibody each reaction

Validation

These antibodies are validated and extensively used in previous publications as listed on manufacturer webpages:
Anti-HMGCR (https://www.novusbio.com/products/hmg-coa-reductase-hmgcr-antibody-jf0981_nbp2-66888)
Anti-Digoxigen-AP (https://www.sigmaaldrich.com/US/en/product/roche/11093274910)
Anti-SREBP2 (https://www.ptglab.com/products/SREBF2-Antibody-28212-1-AP.htm)
Anti-PSCK9 (https://www.abcam.com/products/primary-antibodies/pcsk9-antibody-ab28770.html)
Anti-GAPDH (https://www.sigmaaldrich.com/US/en/product/sigma/g9545)
Anti-CD68 (https://www.bio-rad-antibodies.com/monoclonal/mouse-cd68-antibody-fa-11-mca1957.html?f=purified)
Anti-SR-BI (https://www.novusbio.com/products/sr-bi-antibody_nb400-104)
Anti-Actin (https://www.sigmaaldrich.com/US/en/product/sigma/a2066)
Anti-LDLR (https://www.abcam.com/en-us/products/primary-antibodies/ldl-receptor-antibody-ep1553y-ab52818)
Anti-Scap (https://www.thermofisher.com/antibody/product/SCAP-Antibody-Polyclonal/PA5-28982)
Anti-Insig 2 (https://www.ptglab.com/products/INSIG2-Antibody-24766-1-AP.htm)
Anti-Insig 1 (https://www.ptglab.com/products/INSIG1-Antibody-55282-1-AP.htm)
Anti-SREBP2 (https://www.abcam.com/en-us/products/primary-antibodies/srebp2-antibody-ab30682)

Eukaryotic cell lines

Policy information about cell lines and Sex and Gender in Research

Cell line source(s)	HepG2 cell line (ATCC, HB-8065 ™)
Authentication	This cell line is a common used cell line that was purchased from ATCC. The cell line was not recently authenticated.
Mycoplasma contamination	The cell line used in this study was negative for myoplasma contamination.
Commonly misidentified lines (See ICLAC register)	No cell line used in this study is listed in the ICLAC current register of commonly misidentified cell lines.

Animals and other research organisms

Policy information about studies involving animals; ARRIVE guidelines recommended for reporting animal research, and Sex and Gender in Research

Laboratory animals

7- 10 week old male and female wild-type C57BL/6J (#000664) and LDL receptor-deficient (# 002207) mice were purchased from the The Jackson Laboratory. They were fed a semisynthetic low-fat (4.2% fat) AIN76 diet containing either low cholesterol (0.02% cholesterol; Research Diets, D00110804C) or high cholesterol (0.5% cholesterol; Research Diet, D00083101C). For tsRNA or ASO treatment, mice were treated with tsRNA or ASO by intraperitoneal injections. For atherosclerosis study, mice were either fed a normal chow diet (LabDiet, PicoLab Rodent Diet 5053) for 7 weeks or fed a chow diet for 1 week and then switched to the high cholesterol deit ((0.5% cholesterol; Research Diet, D00083101C) for 6 weeks. All mice were housed in microisolator cages and were provided ad libitum access to diets before the treatment and deionized water in temperature-controlled room (~21°C) with 12 hr light/dark cycle and and humidity ranging from 30-70%.

Wild animals	No wild animals were used in the study.
Reporting on sex	Both male and female mice for were used for the studies to investigate tsRNA-Glu-CTC expression (Fig. 1h, 1i). For the small RNA-seq and treatment studies, male mice were used due to high cost for those experiments and limited resources. All the animal sex information were provided in the manuscript.
Field-collected samples	*For laboratory work with field-collected samples, describe all relevant parameters such as housing, maintenance, temperature, photoperiod and end-of-experiment protocol OR state that the study did not involve samples collected from the field.*
Ethics oversight	Animal experiments were performed in compliance with approved protocol by the Institutional Animal Care and Use Committee of the University of California, Riverside.

Note that full information on the approval of the study protocol must also be provided in the manuscript.

Clinical data

Policy information about clinical studies

All manuscripts should comply with the ICMJE guidelines for publication of clinical research and a completed CONSORT checklist must be included with all submissions.

Clinical trial registration	*Provide the trial registration number from ClinicalTrials.gov or an equivalent agency.*
Study protocol	*Note where the full trial protocol can be accessed OR if not available, explain why.*
Data collection	*Describe the settings and locales of data collection, noting the time periods of recruitment and data collection.*
Outcomes	*Describe how you pre-defined primary and secondary outcome measures and how you assessed these measures.*

Plants

Seed stocks	*Report on the source of all seed stocks or other plant material used. If applicable, state the seed stock centre and catalogue number. If plant specimens were collected from the field, describe the collection location, date and sampling procedures.*
Novel plant genotypes	*Describe the methods by which all novel plant genotypes were produced. This includes those generated by transgenic approaches, gene editing, chemical/radiation-based mutagenesis and hybridization. For transgenic lines, describe the transformation method, the number of independent lines analyzed and the generation upon which experiments were performed. For gene-edited lines, describe the editor used, the endogenous sequence targeted for editing, the targeting guide RNA sequence (if applicable) and how the editor was applied.*
Authentication	*Describe any authentication procedures for each seed stock used or novel genotype generated. Describe any experiments used to assess the effect of a mutation and, where applicable, how potential secondary effects (e.g. second site T-DNA insertions, mosiacism, off-target gene editing) were examined.*
